# Accurate volume image reconstruction for digital breast tomosynthesis with directional-gradient and pixel sparsity regularization

**DOI:** 10.1117/1.JMI.12.S1.S13013

**Published:** 2025-03-07

**Authors:** Emil Y. Sidky, Xiangyi Wu, Xiaoyu Duan, Hailiang Huang, Wei Zhao, Leo Y. Zhang, John Paul Phillips, Zheng Zhang, Buxin Chen, Dan Xia, Ingrid S. Reiser, Xiaochuan Pan

**Affiliations:** a The University of Chicago, Department of Radiology, Chicago, Illinois, United States; b Stony Brook University, Renaissance School of Medicine, Department of Radiology, Stony Brook, New York, United States; c University of Washington, Department of Mathematics, Seattle, Washington, United States

**Keywords:** digital breast tomosynthesis, image reconstruction, dual-energy X-ray tomography, sparsity regularization

## Abstract

**Purpose:**

We aim to develop accurate volumetric quantitative imaging of iodinated contrast agent (ICA) in contrast-enhanced digital breast tomosynthesis (DBT).

**Approach:**

The two main components of the approach are the use of a dual-energy DBT (DE-DBT) scan and the development of an optimization-based algorithm that can yield accurate images with isotropic resolution. The image reconstruction algorithm exploits sparsity in the subject’s directional derivative magnitudes, and it also performs direct sparsity regularization to help confine the reconstruction to the true support of the subject. The algorithm is demonstrated with three sets of simulations in 2D and 3D, and a physical DE-DBT scan. The last of the three simulations employs an anthropomorphic phantom derived from the VICTRE project, testing quantitative tumor imaging with ICA.

**Results:**

The 2D simulations of the algorithm demonstrate accurate and stable image reconstruction. With the first 3D simulation, the proposed algorithm shows the ability to resolve overlapping objects, and with the anthropomorphic phantom, accurate recovery of the irregular ICA distribution in the shape of a tumor model is demonstrated. Applying the algorithm to DE-DBT transmission data of the CIRS BR3D phantom with solid ICA inserts yields images in which the depth-blurring is greatly reduced and the ICA distribution is accurately reconstructed.

**Conclusion:**

The results for the sparsity regularization algorithm applied to DE-DBT show promise, but as the algorithm performance is necessarily subject-dependent, further investigation using subjects with varying complexity in the ICA distribution is required.

## 1 Introduction

Contrast-enhanced digital mammography has been developed for improving sensitivity to early-stage breast tumors that have angiogenic neovasculature,^1^^–^^3^ and this idea is being extended to contrast-enhanced DBT (CE-DBT).^2^^,^^4^^–^^6^ Beyond the ability to possibly improve detection, the volume imaging capability of CE-DBT may also prove useful for the characterization of tumors for evaluating treatment response following neoadjuvant chemotherapy. In addition, the coregistered imaging of 3D enhancement and anatomical information could possibly provide improved guidance for breast biopsy. Both of these clinical tasks rely on the fidelity of the volume imaging of contrast agents to have accurate position information and tumor uptake volume for accurate longitudinal tumor progression characterization. Current CE-DBT suffers from depth blurring that complicates establishing the 3D borders of an enhanced region corresponding to a given tumor volume. The successful outcome of the proposed work would enable accurate tumor volume estimation and could therefore yield significant improvement in the application of CE-DBT for treatment follow-up and breast biopsy.

Accordingly, the primary aim of this work is to develop a practical algorithm that can overcome the depth blurring associated with image reconstruction from data covering a limited angular range (LAR), and adapt it to digital breast tomosynthesis (DBT) imaging. Image reconstruction from LAR data has been a topic of great interest in the literature,^7^^–^^9^ but only recently has it been demonstrated for discrete-to-discrete LAR image models that the associated inverse problem can be solved for angular ranges typical of DBT, i.e., 15 deg to 50 deg scanning arcs.^10^ The algorithm developed by Zhang et al. was motivated by work reported by Xu et al.,^11^ where it was observed that imposing separate sparsity regularization terms on the directional derivative magnitude images was effective at image recovery for LAR scanning. In developing this idea, Zhang et al. derived an optimization-based approach that exploits constraints on the image directional total variation; this formulation has the advantage that it is convex in contrast to previous optimization-based approaches for LAR image reconstruction. Building off of this work, we develop a data-discrepancy-constrained, sparsity regularization algorithm framework that can be applied to DBT scanning data.

In the application to CE-DBT, it is of interest also to isolate and estimate the distribution of iodinated contrast agent (ICA), and accordingly, we seek to develop dual-energy DBT (DE-DBT). In this work, the DE-DBT scan is accomplished by taking two scans at low and high kV settings. A processing pipeline is developed that uses constrained, sparsity regularization for image reconstruction and includes additional post-processing followed by weighted subtraction to obtain the ICA distribution. In Sec. 2, the algorithm theory and application to DBT are described; in Sec. 3, 2D simulations are presented that illustrate the parameter dependence of the algorithm and reconstructed images are shown for a DE-DBT scan of a test phantom containing ICA. Finally, conclusions are presented in Sec. 4.

## 2 Methods for Inverse Problem Studies and 3D Dual-Energy DBT Image Reconstruction

In developing the image reconstruction algorithm for processing the DE-DBT transmission data, the image subtraction method is used to isolate the ICA distribution. Accordingly, the key component in this processing pipeline is the algorithm for reconstructing the 3D volumes from the limited angular range scan configuration of DBT. In this section, the image reconstruction algorithm from LAR data is presented in a 2D framework for the purpose of presenting illustrative 2D inverse problem simulations; generalization to 3D is then explained; and finally, the details of the full processing pipeline for obtaining 3D ICA distributions are presented.

### 2.1 Directional Total-Variation Constrained Optimization

In the idealized LAR image reconstruction problem, the data are modeled as the X-ray transform of the discretized image

g=Xf, (1)

where f is the discretized image array, g is the sonogram, and X is the matrix that encodes fan-beam or cone-beam projection in 2D or 3D, respectively. The z-direction, also referred to as the “depth” direction, is perpendicular to the fixed detector plane in the DBT configuration. The x and y directions span the detector plane and are referred to as “in-plane” directions. For the 2D LAR model, the x- and z-directions are also referred to as “in-plane” and “depth” directions, respectively, even though the subspace perpendicular to z is 1D, and not 2D.

For DBT scanning, typical angular ranges for commercial devices range from 15 deg to 50 deg, and the corresponding algebraic model in Eq. (1) is generally underdetermined, meaning that there is no unique f for a given sinogram g. An important aspect of this linear system, however, is that its ill-posedness depends on the discretization of the image; this is a point that we return to in Sec. 3. Because we are interested in developing quantitative DBT with isotropic resolution, in this work, only square pixels (cubic voxels) are considered. For typical image resolution and LAR scanning, Eq. (1) is underdetermined.

In a previous work, accurate image reconstruction, for useful image resolution, from LAR data was demonstrated by the use of non-smooth convex optimization by Zhang et al.^10^ The specific optimization problem of interest in that work enforced prior information on the directional total variation (DTV)

$$f^{⋆}=\arg\;\min_{f}\{\frac{1}{2}\|g-Xf{\|}_{2}^{2}\;\text{such that}\;\|\partial_{x}f{\|}_{1}\leq \gamma_{x},\|\partial_{z}f{\|}_{1}\leq \gamma_{z}\},$$ (2)

where $\partial_{x}$ and $\partial_{z}$ represent the finite difference approximations to the derivatives in the in-plane and depth directions, respectively; the $\ell_{1}$ terms, $\|\partial_{x}f{\|}_{1}$ and $\|\partial_{z}f{\|}_{1}$, are the DTVs; and the parameters $\gamma_{x}$ and $\gamma_{z}$ are the respective constraint parameters for the DTVs. As least-squares (LSQ) is used for the data fidelity term, this optimization problem is referred to as DTV-constrained LSQ or DTV-LSQ. The use of DTV constraints proves to be more effective than the use of the standard TV constraint, which exploits object gradient sparsity, for accurate image reconstruction.^10^ A heuristic argument for why this is the case is presented in Sec. 3.1. This DTV-constrained optimization is particularly convenient for inverse problem studies with simulated phantoms because the exact DTV values can be readily computed from the test phantom and employed as constraint parameters $\gamma_{x}$ and $\gamma_{z}$. When reconstructing LAR using real DBT data, the DTV constraint parameters need to be determined by other means because their true values are unknown. Accordingly, for the present studies on LAR image reconstruction, we reformulate the optimization problem using a more convenient parameterization and include additional design features that enable adaptation to image reconstruction in DBT with isotropic resolution.

### 2.2 Data-Discrepancy Constrained, Sparsity Regularization

For LAR image reconstruction considered here, we reverse the roles of the constraints and the objective function and include additional sparsity-exploiting terms. The optimization problem that specifies the image reconstruction in this work is

$$\begin{array}{l} f^{⋆}=\arg\;\min_{f}\{\alpha_{x}\|\partial_{x}f{\|}_{1}+\alpha_{y}\|\partial_{y}f{\|}_{1}+\alpha_{z}\|\partial_{z}f{\|}_{1}+\beta \|f{\|}_{1}\\ \text{such that}\;\|R[c](g-Xf){\|}_{2}\leq \epsilon \cdot \sqrt{\text{size}(g)}\;\text{and}\;f\geq 0\}, \end{array}$$ (3)

where the parameters $\alpha_{x}$, $\alpha_{y}$, and $\alpha_{z}$ control the admixture of directional-TV terms (for the 2D case the y-directional TV term is dropped); β controls the strength of the $\ell_{1}$-norm penalty term; ε is a data-discrepancy constraint parameter specified as a root-mean-square-error (RMSE); and the linear operator R[c] encodes sinogram filtering with the square root of the Hanning filter with cutoff parameter c, specified as a fraction of the Nyquist frequency. The size(·) operator yields the number of elements in the vector argument.

If β is set to zero, the y-directional TV term is ignored, and R[c] is replaced by the identity matrix, this optimization problem yields equivalent solutions to the DTV-LSQ optimization problem in Eq. (2), i.e., for given $\gamma_{x}$ and $\gamma_{z}$ with DTV-LSQ, there are corresponding α and ϵ in Eq. (3) that yields the same solution, where $\alpha_{x}=\alpha$ and $\alpha_{z}=2-\alpha$. This equivalence is established through the Lagrangian of both optimization problems. We find the parameterization in Eq. (3) more convenient for real data situations because the ϵ parameter choice is guided by the noise level in the data and the useful range of α is restricted to 1<α<2. This range for α for LAR scanning results from the fact that the in-plane DTV constraint for DTV-LSQ is the main constraint responsible for allowing accurate image recovery, which is seen in the heuristic argument of Sec. 3.1.

The optimization in Eq. (3) has two additional features that assist in the application of image reconstruction from LAR scanning data. The $\ell_{1}$-norm penalty controlled by β encourages pixel/voxel sparsity in the reconstructed image. As LAR image reconstruction generally blurs the image in the depth direction, the image values tend to spread beyond the image boundary in z. The $\ell_{1}$-penalty assists in restricting the reconstructed image to the true support of the object. The square root of the Hanning filter R[c] in the data-discrepancy constraint has two roles: (1) the ramp-like filtering acts as a preconditioner,^12^ and (2) due to the high-pass filtering, data-discrepancies at low spatial frequencies are de-emphasized reducing artifacts due to X-ray scatter and imperfect estimation of the $I_{0}$ blank scan fluence needed to process the transmission data into sinograms. The cut-off parameter c proves useful for controlling errors due to image discretization.

Because Eq. (3) involves multiple sparsity regularization terms, this optimization is generically referred to as constrained, sparsity regularization. In Sect. 3, when β is set to zero, we will refer to this optimization as constrained, DTV min, or simply DTV min. When β>0, we will refer to this optimization as constrained, L1-DTV min, or simply L1-DTV min. We note that when α=1, the directional TV penalties are all of the same strength and the terms involving the image gradient become the standard TV penalty. The posed optimization for constrained, sparsity regularization is convex but non-smooth due to constraint and $\ell_{1}$-norm-based penalty terms. To solve this optimization problem, we use the primal-dual hybrid gradient (PDHG) algorithm of Chambolle and Pock.^13^^,^^14^

Due to the fact that iterative image reconstruction for LAR configurations can converge rather slowly, we adopt a couple of strategies that seem to improve the convergence rate for the problems of interest. The first strategy is the use of R[c]-filtering in the data-discrepancy constraint in Eq. (3); we note that the use of the filtering in this way differs slightly from the original reference Wang et al.,^12^ in which they used the R[c]-filtering only for preconditioning by applying this filter to the Lagrange multiplier term in an alternating directions method of multipliers framework. In this way, Wang et al. do not modify the data fidelity term. In our usage, applying this filter to the data discrepancy effectively alters the data fidelity measure, which for our application has the benefit of de-emphasizing low spatial-frequency inconsistencies. Readers interested in preconditioning without altering the data fidelity term are referred to Wang et al.^12^ The second strategy that we have found effective is the use of the predictor-corrector modification to the PDHG algorithm developed in Algorithm 4 in He and Yuan.^15^ This proposed simple modification to PDHG is analogous to relaxation in the algebraic reconstruction technique, and it requires the specification of a relaxation parameter ρ∈(0,2), and for this work, we choose ρ=1.75. For the results presented in this work, 2000 iterations of the PDHG algorithm are performed, which we find is sufficient for accurate numerical convergence of the sparsity regularization optimization problem. For clinical applications, early stopping and sequential processing methods, such as ordered subsets, can substantially improve algorithm efficiency.

### 2.3 Inverse Problem Studies in 2D

To gain some understanding of the parameter dependence of constrained, sparsity regularization, we conduct inverse problem studies for ideal noiseless data in a 2D LAR scanning configuration. The varied parameters are the penalty parameters α and β, and we also consider a variation of the number of pixels. For the image representation, square pixels are employed because the goal is to achieve isotropic resolution, but increasing the pixel size (worsening resolution) is expected to improve the problem conditioning. Note that in the ideal data studies, there is no discretization error because the sinogram data is generated from a phantom pixelized on the same grid used for the image reconstruction. We conduct the inverse problem studies in a slightly different manner than what was reported in Zhang et al.;^10^ instead of enforcing an equality constraint on the data, we loosen the data constraint to a small ϵ value. In this way, a measure of solution stability is obtained by taking the ratio of the reconstruction image error to ϵ.

For the comparison algorithm in the inverse problem studies, Tikhonov regularized least-squares (LSQ-Tik) optimization is used in a comparable data-discrepancy-constrained form

$$f^{⋆}=\arg\;\min_{f}\{\frac{1}{2}\|\nabla f{\|}_{2}^{2}\;\text{such that}\;{\left\|g-Xf\right\|}_{2}\leq \epsilon \cdot \sqrt{\text{size}(g)}\},$$ (4)

where ∇ is the finite differencing approximation to the image gradient. To solve LSQ-Tik, the roughness penalized LSQ form is solved using conjugate gradients. The penalty parameter is then adjusted until the data discrepancy is equal to the constraint value ϵ. In this way, the results are directly comparable with those of constrained, sparsity regularization.

The phantom used for the 2D studies is a computer-simulated object representing structures based on a 2D slice of a breast. The distribution of the glandular tissue is generated by thresholding a realization of power-law noise, and randomly located specks are included to model microcalcifications.^16^^,^^17^

### 2.4 Simulation Studies and Image Reconstruction in 3D

To tune algorithm parameters and adapt the sparsity regularization algorithm to DBT imaging, simulation studies are performed with two computer-simulated test phantoms. The first phantom has the overall form of a compressed breast, and it is composed of analytic geometric objects such as rectangular slabs, spheres, and semi-ellipsoids.^18^ The second phantom is derived from the virtual anthropomorphic breast model developed at the U.S. Food and Drug Administration (FDA) as part of the Virtual Imaging Clinical Trials for Regulatory Evaluation (VICTRE) project.^19^ The test object derived from the VICTRE phantom focuses on the CE-DBT application and consists only of a contrast-enhanced tumor and background enhancement of glandular tissue at a level of 20% of the tumor value.

For both the analytic and anthropomorphic phantoms, continuous projection is modeled. In the former case, the analytic projection of the geometric objects comprising the phantom is known, and in the latter case, the digital phantom is defined on a 50-micron grid, which is much smaller than the voxel size used for image reconstruction. Continuous object modeling allows us to test discretization error, which is one of the leading sources of data inconsistency when large voxels are used for the reconstruction volume. As the voxel size is increased, fewer voxels are needed to represent the same volume and the LAR image reconstruction becomes more well-posed. On the other hand, larger voxels lead to greater discretization error. The use of noiseless, continuous projection data allows the study of the trade-off between well-posedness and discretization error and guides the sparsity regularization parameter selection.

Naturally, it is important to consider other physical factors that can have an impact on DBT image reconstruction algorithms such as noise, X-ray scatter, and beam polychromaticity. It is for this reason that we apply the proposed sparsity regularization algorithm to DE-DBT data acquired with the physical CIRS BR3D phantom with ICA inserts.

### 2.5 Processing Pipeline for Dual-Energy DBT Scanning

To apply constrained, sparsity regularization to image reconstruction for ICA imaging in DE-DBT a number of pre- and post-processing operations are performed. In this work, the only pre-processing performed involves measuring flood field scans, $I_{0}$, at the low and high X-ray source kV settings. The sinogram for both scans is obtained from the transmission data I by the standard Beer-Lambert model

$$g=-\ln(I/I_{0}).$$ (5)

Note that using this assumption, beam-hardening is not accounted for.

The sinogram data are subsequently reconstructed by constrained, sparsity regularization and two image volumes $f_{\text{low}}$ and $f_{\text{high}}$ are obtained from the sinograms for the low and high kV settings, respectively. These images are susceptible to low spatial-frequency artifacts that result from X-ray scatter, beam-hardening, and possibly errors in the $I_{0}$ estimates. To combat these artifacts, 2D polynomial fitting is performed slice by slice in the x−y planes by solving the following optimization

$$a^{⋆}=\arg\;\min_{a}\|m\cdot (s-Pa){\|}_{1},\;b=Pa^{⋆},$$ (6)

where s is the input raw slice image, P is a matrix where each column is a polynomial basis function, a is a vector of the unknown polynomial coefficients, m is an image mask determined by a gray value threshold, and b is the estimated background gray values for the slice s. The use of the $\ell_{1}$-norm in Eq. (6) allows for the fit of Pa to s to not be overly influenced by outliers. Because it is difficult to estimate a mask m that exactly conforms to the object support in the slice s, large errors are expected near the true object boundary; the use of the $\ell_{1}$-norm reduces the sensitivity of the fitting to these errors. The algorithm used for solving Eq. (6) is iteratively reweighted LSQ.^20^ Stacking the background slices b and masks m yields the background and mask volumes $f_{b}$ and $f_{m}$, respectively. The display volume, $f_{d}$, is obtained by dividing by the background volume and masking

$$f_{d}=f_{m}\cdot \frac{f}{f_{b}}.$$ (7)

To obtain the desired ICA image, weighted subtraction of the high and low kV images is performed

$$f^{\left(\mathrm{ICA}\right)}=(f_{d}^{\left(\text{high}\right)}-1)-w\cdot (f_{d}^{\left(\text{low}\right)}-1),$$ (8)

where the weight w is determined in this work by visual inspection, i.e., the value w that minimizes the non-ICA background is selected. In Eq. (8), one is subtracted from both display volumes because the background gray values in both images are expected to be distributed symmetrically about one.

### 2.6 DE-DBT Acquisition and Physical Phantom Studies

**Fig. 1:**
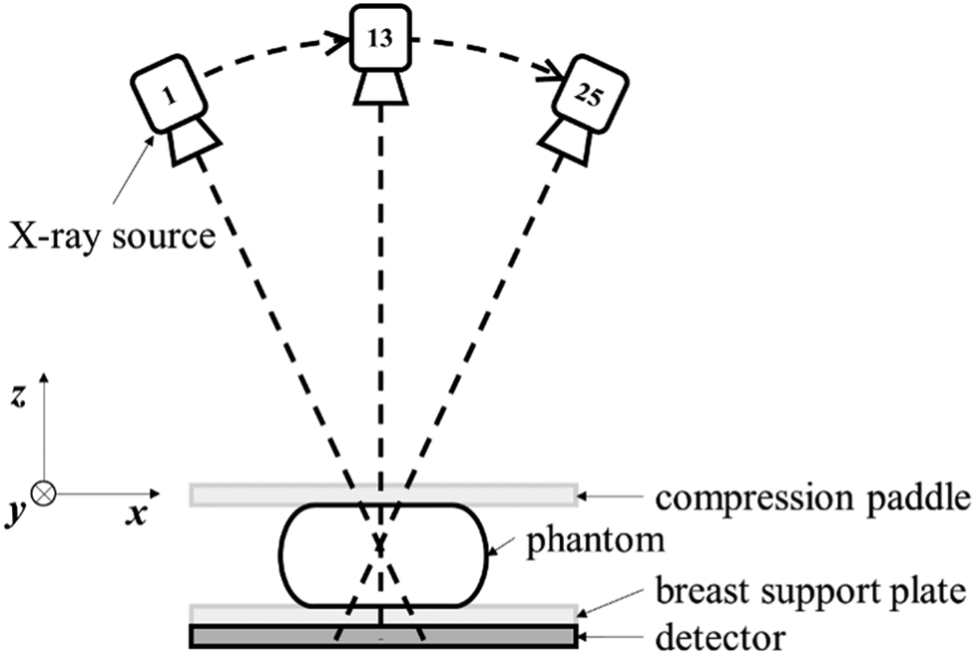
Schematic of DBT scan geometry.

A DE-DBT scan consists of two separate DBT acquisitions, taken sequentially with a research Siemens Mammomat DBT system. Both the low-energy (LE) and high-energy (HE) DBT acquisitions utilize a tungsten anode. The LE-DBT is acquired at a source potential of 30 kV with Al filtration, and the HE-DBT at 49 kV with Ti filtration. As a result, the X-ray spectra straddle the K-edge of iodine, which is located at 33.2 keV. Automatic exposure control results in exposures of 75 and 51 mAs for LE- and HE-DBT, respectively.^2^ For each DBT acquisition, the tube travels in continuous motion, spanning an angular range of ∼50  deg with 25 projections at ∼2  deg intervals. Because the dual-energy scan involves two passes of the X-ray tube, a total of 50 projections are acquired. A schematic of the scanner geometry is shown in Fig. 1. The actual scan geometry varies slightly from this plan, and the accurate projection view angles are stored in the projection DICOM files. The configuration is a half-cone illumination with the scan arc directly in line over the long edge of a 3584×2816  pixel flat-panel detector, which remains fixed during the scan. The native detector pixel size is ${\left(85\;\text{microns}\right)}^{2}$.

The object scanned is a 5-cm-thick BR3D phantom (Model 020, CIRS Inc. Norfolk, VA). This phantom has structural complexity where two plastics of density similar to adipose and glandular tissue are swirled together. The phantom comes in 1-cm-thick semi-circular slabs, and for this work, the central slab of five contains solid ICA inserts. The ICA inserts vary in size and concentration; the diameters are 2, 3, 5, and 8 mm, the cylindrical height equals the diameter, and the iodine concentrations are 1, 2, 3, and 5  mg/mL.

## 3 Results

The results illustrate the application of constrained, sparsity regularization to image reconstruction from LAR data. The inverse problem-oriented results compare no sparsity regularization with TV min, DTV min, and L1-DTV min. In addition, the impact of the image size on the accuracy of image reconstruction is also shown. Constrained, sparsity regularization is then applied to ICA imaging with DE-DBT.

### 3.1 Tikhonov-Regularized, Least-Squares

To illustrate the image reconstruction challenge presented by LAR data, image reconstruction is performed with a 2D simulation using a scan configuration similar to that of the Siemens DBT system. The scan configuration consists of 25 fan-beam projections covering a 50° arc. The setup is similar to CT in that the detector and X-ray source rotate about the central point in the image with the isocenter-to-source distance set to 50 cm and the source-to-detector distance set to 100 cm. The detector is taken to be a linear array of 1024 detector bins with a total length of 20.1 cm. This length is chosen so that the field of view is the largest inscribed circle in the 10  cm×10  cm image. For this initial reconstruction, the image array is taken to be 512×512  pixels. The reason why a CT-style setup is chosen is that we aim to focus on the issue of LAR scanning without the additional complication of having a shifting field-of-view inherent to a fixed detector set-up typical of DBT.

**Fig. 2:**
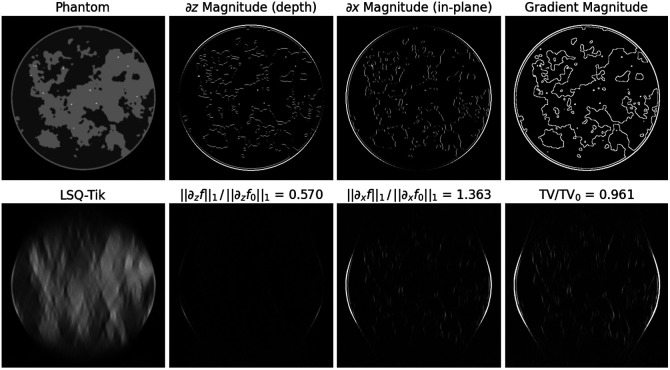
LSQ-Tik reconstructed image along with gradient magnitude images. Image reconstruction of 50-deg scanning data using LSQ-Tik with ϵ=0.001. The top row shows the test phantom along with the magnitude images of the z-derivative, x-derivative, and the gradient. The sum over the gradient magnitude image (GMI) is the TV norm. The bottom row shows the LSQ-Tik reconstructed image along with the same derivative/gradient magnitude images. The corresponding DTV and TV values are reported as ratios with the ground truth DTV and TV values.

As an inverse problem, it is already clear that there is not a unique solution to Eq. (1) because the number of unknown pixel values is $512^{2}$ (i.e., 262,144), which is much larger than the number of measured ray integrals, 25 views by 1024 bins (i.e., 25,600). In addition, the conditioning of X is worsened by the limited scanning arc. The use of LSQ-Tik yields the image shown on the lower left panel in Fig. 2. The ill-conditionedness of this image reconstruction problem is evident as the reconstructed image is severely distorted with the well-known depth blurring inherent to DBT.

With an eye toward sparsity regularization, it is instructive to inspect the derivate magnitude images. The $\left|\partial_{z}f\right|$ image has very few significant non-zero values, and few of the edges seen in the phantom $\left|\partial_{z}f_{0}\right|$ image are reproduced. This is a consequence of the fact that these edges are not “visible” in a microlocal sense for this LAR configuration.^8^ The $\left|\partial_{x}f\right|$ image, on the other hand, does reproduce many of the in-plane edges from the ground truth; this is why DBT has utility for medical imaging. The gradient magnitude image (GMI) resembles the $\left|\partial_{x}f\right|$ image because the values of $\partial_{z}f$ are much smaller than those of $\partial_{x}f$.

The results in Fig. 2 also provide a heuristic argument as to why DTV-constrained LSQ can be more effective than TV-constrained LSQ. Because the $\partial_{z}f$ values are small compared with ground truth, the GMI values are also smaller compared with ground truth. Because the TV-norm is the sum over the GMI, the LSQ-Tik reconstructed image has a TV smaller than the ground truth. As a result, imposing a TV inequality constraint with the constraint value set to the ground truth has no effect. On the other hand, the x-DTV, $\left\|\partial_{x}f\right\|$ has a value greater than the ground truth and imposing a constraint on x-DTV restricting it to values less than or equal to the ground truth will have an effect. From these observations, it is clear that the x-DTV constraint in DTV-LSQ, Eq. (2), plays a more important role than the z-DTV constraint.

There is also a corresponding heuristic for DTV-min. Given that the phantom has similar structural complexity in $\partial_{z}f$ and $\partial_{x}f$, the ground truth values of x-DTV and z-DTV should be comparable. The LSQ-Tik result however results in x-DTV much greater than z-DTV. Thus, when selecting an α for DTV-min, putting a greater weight on the x-DTV term would bring the two norms closer to the actual ratio of x-DTV to z-DTV and accordingly α should be selected in a range (1,2).

### 3.2 Constrained, Sparsity Regularization

Various forms of constrained, sparsity regularization are applied to the same image reconstruction problem of Sec. 3.1. The sparsity regularization algorithms considered are TV min, DTV min, and L1-DTV min. For TV min, the optimization problem solved is Eq. (3) with α=1 and β=0. For DTV min, Eq. (3) is solved for β=0 and varying α; the α value that minimizes the image RMSE is chosen, which is possible only in the context of simulation where the ground truth is known. Likewise, for L1-DTV min, both α and β are optimized on the image RMSE.

**Fig. 3:**
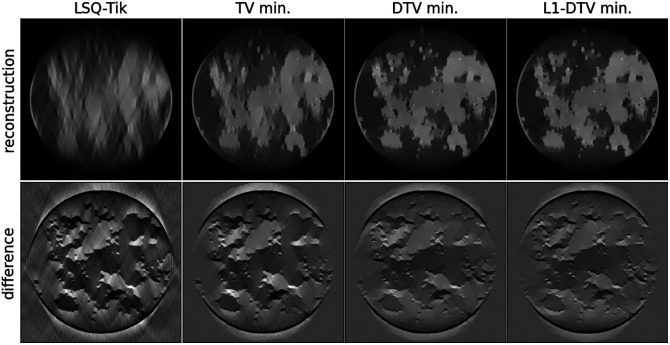
Sparsity regularization with 51200D7512 phantom. Reconstructed images comparing LSQ-Tik with various forms of constrained, sparsity regularization using a 512×512 grid of $(0.2\;\mathrm{mm}{)}^{2}$ pixels for the test phantom and reconstructed image array. The top row shows the reconstructed images in a grayscale of [0,1.5], and the bottom row shows the difference between the reconstructed images and the ground truth in a grayscale of [−0.5,0.5].

The results for the setup in Sec. 3.1, using a 512×512 image array, are shown in Fig. 3. As expected the optimal α, which is found to be 1.7, falls in the range (1,2). Visually, there is a clear improvement in the image reconstruction accuracy in going from LSQ-Tik to TV min and in going from TV min to DTV min. The use of L1-DTV min does not appear to improve image reconstruction accuracy significantly over DTV min.

**Fig. 4:**
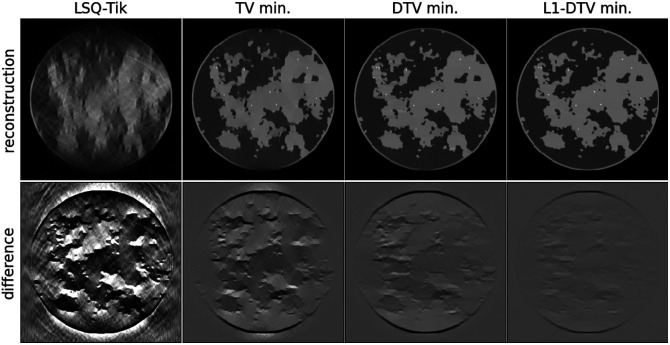
Sparsity regularization with 256×256 phantom. The same as Fig. 3 except that the test phantom and image array are on a 256×256 grid of $(0.4\;\mathrm{mm}{)}^{2}$ pixels, and the difference in image grayscale is [−0.25,0.25].

**Fig. 5:**
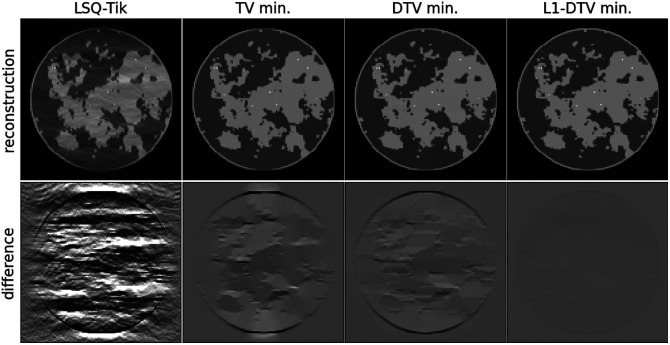
Sparsity regularization with 128×128 phantom. The same as Fig. 3 except that the test phantom and image array are on a 128×128 grid of $(0.8\;\mathrm{mm}{)}^{2}$ pixels, and the difference in image grayscale is [−0.1,0.1].

Another significant parameter of the algebraic model in Eq. (1) is the number of pixels, or equivalently, the pixel size. Reducing the number of pixels should improve the conditioning of the image reconstruction problem and more accuracy is expected. We repeat the same LAR reconstruction problem, using image arrays of size 256×256 and 128×128, which have a total pixel number of 65,536 and 16,384, respectively. The corresponding results are shown in Figs. 4 and 5. Interestingly, the optimal α value for DTV min increases as the image array size decreases; α=1.9 and 1.95 for the cases of 256×256 and 128×128 images, respectively. As a general trend, the image reconstruction accuracy does indeed improve with the smaller image arrays, and visual image recovery improves going from left to right in these sets of results. The L1-DTV min result for the 128×128 image has a barely perceptible error in the shown grayscale range [−0.1,0.1].

We note that under the conditions of this inverse problem study, exact recovery is not possible because the data discrepancy is constrained to an RMSE of ϵ=0.001 instead of zero. To have a quantitative sense of the image reconstruction accuracy, the image RMSEs for all forms of constrained, sparsity-regularization, and image array sizes are shown in Table 1. By far, the lowest image RMSE is obtained for the 128×128 image size using L1-DTV minimization. As the image RMSE is only 1.4 times the data RMSE, there is some measure of stability for image reconstruction in this case.

**Table 1:** Image RMSE for various forms of constrained, sparsity regularization. Image RMSEs for various forms of sparsity regularization and for different image array sizes. For each result, the data discrepancy is $\epsilon =1\times 10^{-3}$.

Phantom size	512×512	256×256	128×128
TV min	$123\times 10^{-3}$	$50\times 10^{-3}$	$19\times 10^{-3}$
DTV min	$109\times 10^{-3}$	$28\times 10^{-3}$	$8.4\times 10^{-3}$
L1-DTV min	$102\times 10^{-3}$	$10\times 10^{-3}$	$1.4\times 10^{-3}$

From these inverse problem studies, it is clear that to achieve isotropic resolution with a 50 deg scanning arc, the use of L1-DTV min will outperform the other studied forms of sparsity regularization. Furthermore, the pixel size of the image array needs to be substantially larger than that of the detector bin widths. We note that the inverse problem studies here do not consider the impact of discretization error, which is expected to get worse with larger pixels. Nevertheless, large pixels of at least a factor of four times the detector bin size are needed, because the image errors for the smaller pixel cases in Table 1 range from a factor of 10 to 100 larger than the data RMSE.

### 3.3 Simulation Studies for L1-DTV min in 3D DBT

For the next set of results, two 3D computer-simulated breast phantoms are used that model the geometry of a compressed breast. The scan configuration is taken to be that of the Mammomat scanner, which is illustrated in Fig. 1. The projection data are generated by continuous line integration, and accordingly, discretization error is present. No other physical factors, such as noise, scatter, or beam hardening, are modeled. The analytic phantom is used to determine the algorithm parameters: voxel width; regularization parameters $\alpha_{x}$, $\alpha_{y}$, $\alpha_{z}$, and β; the cut-off frequency parameter c used in the R[c]-filter; and the data discrepancy ε. The determination of these parameters is based on minimizing the volume RMSE between the reconstruction and the analytic phantom. For subsequent image reconstruction results, all of the same parameter settings are used except for ϵ. The parameter ϵ needs to be adjusted for each result because its setting reflects the level of data inconsistency present in the projection data.

**Fig. 6:**
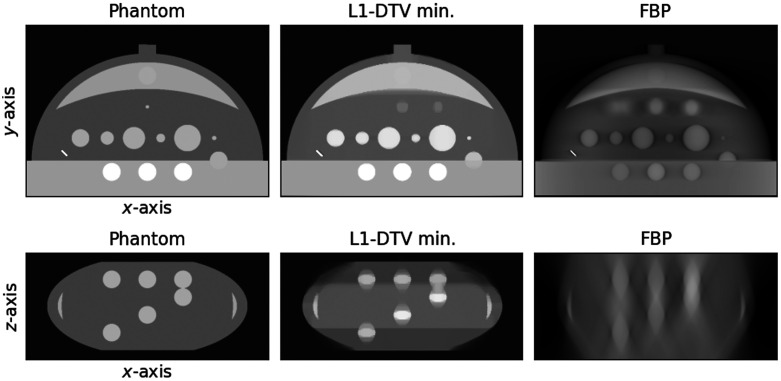
Reconstructed images with the analytic breast phantom. The top row shows reconstructed in-plane images for L1-DTV min and FBP along with the phantom for reference. The bottom row shows a depth plane image for a slice that contains spherical objects that test the z-resolving capability of the image reconstruction algorithms.

**Fig. 7:**
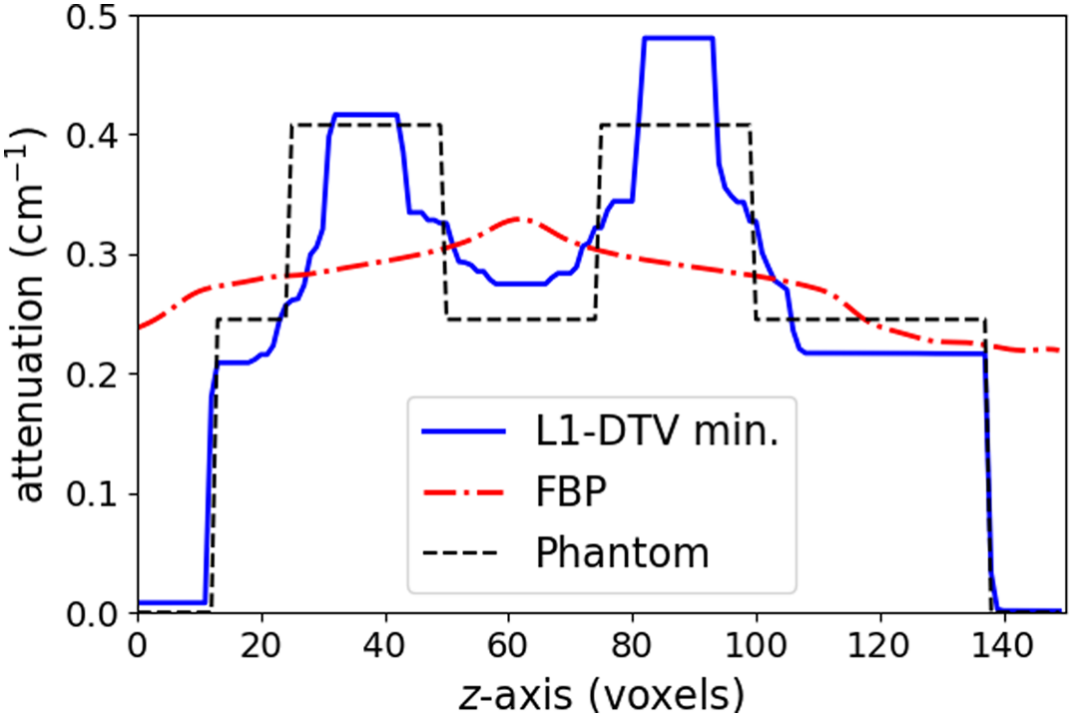
Depth profile for reconstructed images shown in Fig. 6. The plotted profiles are shown for a vertical line traversing the middle set of spheres shown in the bottom row of Fig. 6.

The results of the parameter optimization for L1-DTV min with the analytic breast phantom are shown in Fig. 6. The FBP image is shown alongside the L1-DTV result for reference. Although there are visible differences between the L1-DTV image and the phantom, L1-DTV can recover the gross features of the test object. In particular, in the depth slice shown in the second row of Fig. 6, the L1-DTV image has good recovery of the phantom shape and resolves the overlapping spherical signals. For a quantitative comparison, a line profile is plotted in the z-direction for a line that goes through the middle of two overlapping spherical signals in Fig. 7. The FBP result illustrates the difficulty of obtaining quantitatively accurate DBT volumes; the L1-DTV result shows quantitative accuracy at the level of ∼15%. In minimizing the image RMSE for this phantom, the parameter settings are a voxel width of 370 microns (four times the detector pixel width); $\alpha_{x}=1$, $\alpha_{y}=3.5$, $\alpha_{z}=0.05$, and β=0.02; cut-off frequency c=0.25; and ϵ=0.006. We point out that there is an overall constant multiplier of the α and β parameters that will not impact the solution. Thus, $\alpha_{x}$ is fixed to the value 1, and the remaining six parameters are optimized on a coarse grid. For subsequent studies, all parameter settings are fixed to these determined values except for ϵ, which is adjusted on a case-by-case basis.

The next set of results uses a phantom derived from the anthropomorphic VICTRE phantom. As the target application is volume imaging of an ICA distribution in the breast, we construct a phantom with a contrast-enhanced tumor and include a low-level enhancement of background glandular tissue. The DBT projection data for such an object could be obtained by dual-energy decomposition in the sinogram domain. For this experiment, the assumption is that the ICA sinogram is extracted exactly and we focus only on the image reconstruction for LAR data of this ICA distribution. The confounding factors for this image reconstruction problem are the LAR data itself, and discretization error as the ICA distribution phantom is defined for a much finer voxel grid than the image reconstruction volume.

**Fig. 8:**
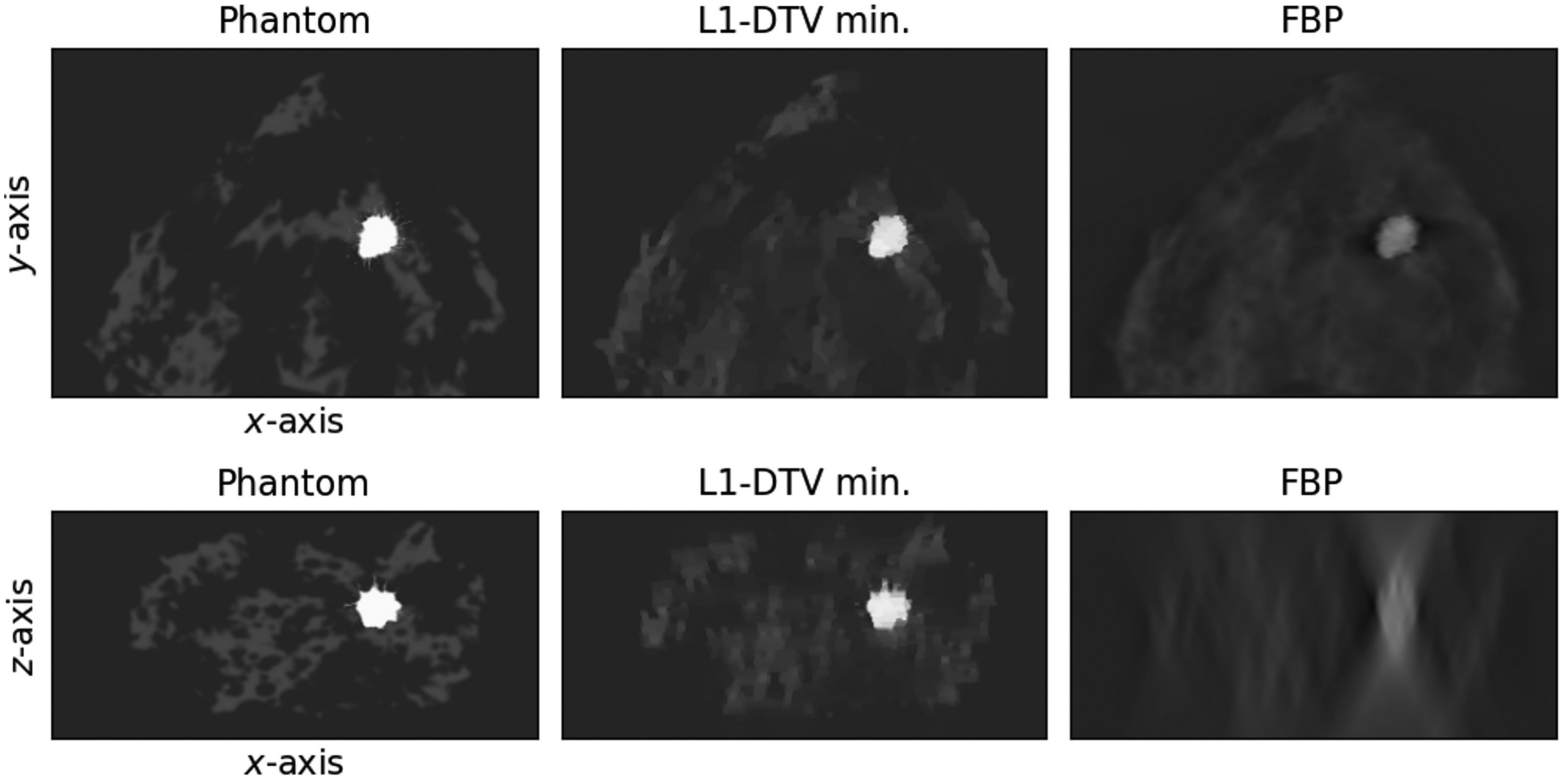
Reconstructed images of the VICTRE ICA distribution phantom. The top and bottom rows show reconstructed in-plane and depth plane images, respectively, for L1-DTV min and FBP along with the phantom for reference. The selected planes for both sets of images are chosen so that they cut through the contrast-enhanced tumor.

**Fig. 9:**
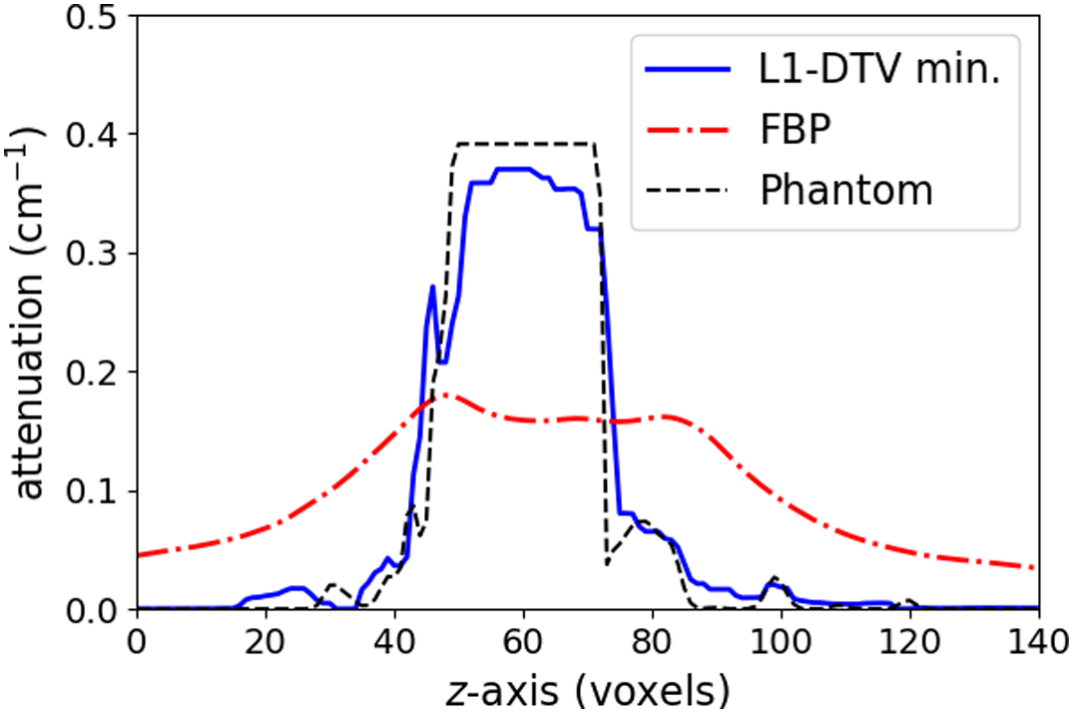
Depth profile for reconstructed images shown in Fig. 8. The plotted profiles are shown for a vertical line traversing the middle of the contrast-enhanced tumor in the bottom row of Fig. 8.

In obtaining the resulting images shown in Fig. 8, only the parameter ϵ is returned to $\epsilon =4\times 10^{-3}$ using minimum image RMSE. From the slice images, the outline of the contrast-enhanced tumor is recovered fairly well; in particular, the depth plane images show that the typical depth blurring seen in the FBP image is greatly reduced in the L1-DTV image. The degree of quantitative accuracy can be appreciated in the z-direction line profiles shown in Fig. 9.

An important factor in the success of sparsity regularization techniques is the dependence on the scanned object structure. The extended distribution of the analytic phantom is more challenging for L1-DTV min than the confined tumor distribution of the VICTRE ICA phantom. For the latter results, the extended and structured low-level background ICA distribution is not as well recovered as the contrast-enhanced tumor. Fortunately, the presence of the background ICA distribution does not appear to interfere with the reconstruction of the tumor, which indicates that L1 DTV min may prove useful for the purpose of follow-up imaging of tumors with dual-energy CE-DBT.

### 3.4 DBT Slice Image Background Fitting

For applying constrained, sparsity regularization to ICA imaging in DE-DBT, low, and high kV scans are acquired of the CIRS BR3D phantom with ICA cylinders, as described in Sec. 2.6. Based on the simulation studies of Secs. 3.2 and 3.3, the form of constrained, sparsity regularization chosen is L1-DTV min, and the reconstruction volume consists of voxels four times the width of the detector pixels; namely, we select the voxels to be 340 microns wide. All reconstruction parameters remain the same as those obtained with the analytic phantom simulation except for the data discrepancy-constraint parameter ϵ. Because ground truth is not known, ϵ is tuned visually, and for the high kV data set, we selected a value of ϵ=0.0085. Because the dual-energy processing for this work involves image subtraction, we tuned ϵ to a value of 0.011 for the low kV data set, which results in a noise level that is visually similar to that of the high kV image.

**Fig. 10:**
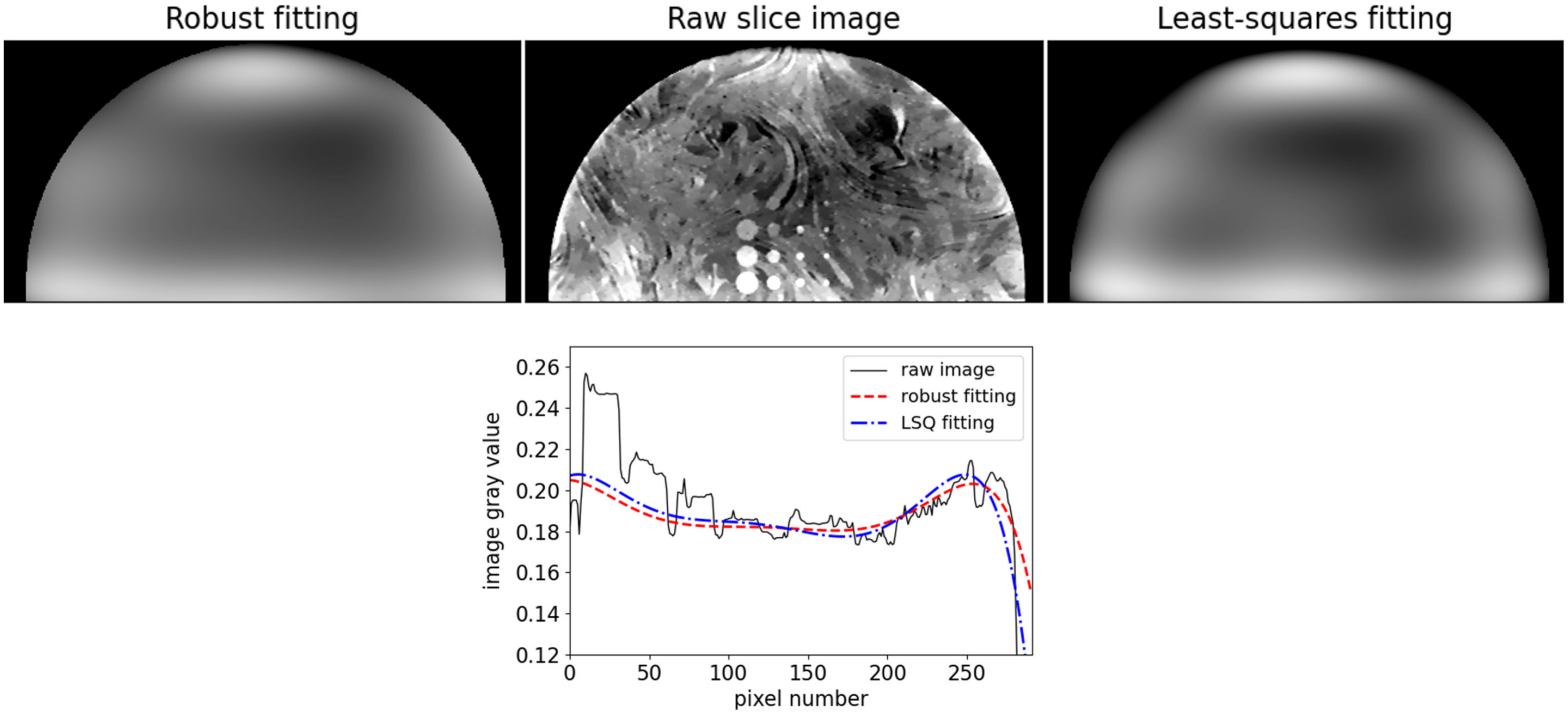
Slice background polynomial fitting with robust and LSQ estimation. The top row illustrates background polynomial fitting for removing the low spatial-frequency artifacts in the raw reconstructed images. The middle panel shows the raw L1-DTV min slice image for the high-kV scan data for a slice that intersects all of the ICA objects. The left panel shows a robust polynomial fit using Eq. (6) to find the coefficients of a 7th-degree 2D polynomial. The right panel shows the LSQ fit for the same polynomial. The bottom row plots image and background fit profiles in the y-direction on a line that passes through the middle of the 8-mm ICA cylinders.

The low and high kV datasets are processed into sinogram estimates and reconstructed with L1-DTV min and FBP. An example raw slice image from the L1-DTV high kV scan is shown in the center panel of Fig. 10. Because the X-ray spectrum for this acquisition is mainly above the iodine K-edge, the ICA objects are clearly visible. Also clearly visible are low spatial-frequency artifacts that make it challenging to window/level the slice image. For DE imaging, these artifacts are particularly confounding because the form of these artifacts is different for the low and high kV scans. As a result, DE subtraction is not possible without first removing these artifacts.

Shown in the left panel of Fig. 10 is the estimated background variation obtained with a 7th-degree polynomial fit, using robust fitting formulated in Eq. (6). To perform the fit, the support of the phantom image is estimated by thresholding, including pixels with a gray value of 10%, of the maximum gray value, or greater. The fit is performed for pixels only within this estimated support. For comparison, the background fit using standard LSQ is shown in the right panel of Fig. 10. LSQ fitting involves replacing the $\ell_{1}$-norm in Eq. (6) with the square of the $\ell_{2}$-norm. Robust fitting is advocated for here because it is less sensitive to outliers. In this application, outliers can originate from pixels beyond the border of the breast that may be within the estimated support, or they can result from high-contrast true structures.

In visually inspecting the fit backgrounds, robust fitting appears to yield a background estimate closer to the variations seen in the raw slice image. Quantitatively, the advantage of robust fitting over LSQ fitting is seen in the profile plot shown in Fig. 10. The curve corresponding to LSQ fitting is more influenced by the high-contrast 8-mm ICA cylinder as this curve is pulled to higher values than that corresponding to robust fitting. Likewise, the LSQ curve is pulled down further than the robust fitting curve at the phantom border on the right side of the plot.

This background fitting is performed also on the low kV image, but it was found necessary to use a 9th-degree polynomial for this case. Both FBP volumes also have their backgrounds estimated using the same robust fitting methodology.

### 3.5 DE-DBT Reconstructed Images

Having found the background variation of the DBT slice images in Sec. 3.4, all slices are divided by the corresponding polynomial fit background as described in Eq. (7). In this way, the reference gray value for the background is one for all slices. To isolate the ICA signals, weighted subtraction of the low and high kV post-processed images is performed as specified in Eq. (8). The weights are chosen by visual inspection, and for L1-DTV min and FBP, they are both found to be 0.8.

**Fig. 11:**
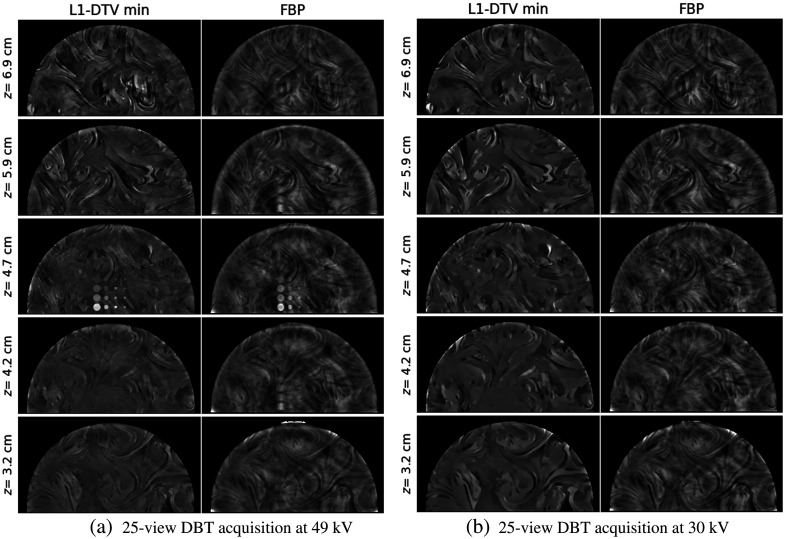
L1-DTV min reconstructed images for DE-DBT. (a) Comparison of post-processed L1-DTV min and FBP slice images for the high kV scan. The slices are ∼1  cm apart in depth, matching the spacing of the BR3D phantom slabs. The grayscale window for both sets of results is [0.8, 1.3]. (b) The corresponding images for the low kV scan. The z coordinates of the slices measure the distance from the detector.

The resulting slice images for the low and high kV data set and the ICA distributions are shown in Fig. 11. The slice separation is chosen so that slice images of each of the BR3D slabs are selected; the slice for the middle slab is chosen so that it intersects with all of the ICA objects. The different background structures for each slab are well-resolved in the L1-DTV min images, whereas the FBP images show the usual interference of structures above and below the viewing plane. Of particular note, the ICA objects are confined to the middle slice for L1-DTV, and the depth blurring of these signals is evident in the FBP images. The low kV images show a similar background structure as the high kV images, but the ICA objects are nearly invisible because the corresponding X-ray beam spectrum is below the k-edge of iodine.

**Fig. 12:**
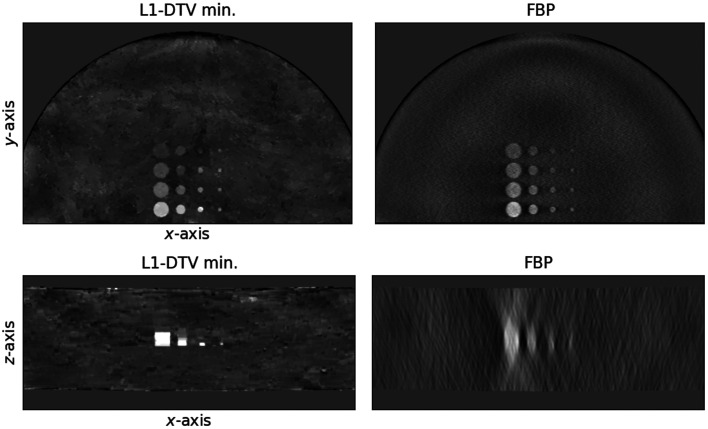
Weighted subtraction images. The top row shows the weighted subtraction images for the z=4.7  cm plane shown in Fig. 11. The bottom row shows a depth plane cutting through the row of ICA objects with the highest iodine concentration.

The weighted subtraction images, isolating the ICA distribution, are displayed in Fig. 12. The depth plane image results for L1-DTV min show that this algorithm can greatly reduce the depth blurring seen in the FBP images. The background subtraction seems to be cleaner for the FBP images than it does for L1-DTV min; this is a side-effect of the nonlinear nature of sparsity regularization. It is expected, however, that the use of dual-energy sinogram processing will mitigate this issue because L1-DTV min would be applied directly to the ICA sinogram. This result demonstrates the potential utility of L1-DTV min for quantitative mapping of ICA distributions. Further investigation, however, is needed to explore the ICA reconstruction accuracy dependence on the complexity of the ICA distribution.

## 4 Conclusion

In this work, we have developed an image reconstruction algorithm that has the potential to achieve quantitative imaging in DE-DBT with isotropic resolution. The main development that enables this capability is the formulation of constrained, sparsity regularization. This optimization framework is an extension of our previous work on the DTV-constrained LSQ algorithm, which has proven useful for solving the algebraic inverse problem associated with image reconstruction from LAR data. The present algorithmic framework includes additional pixel/voxel sparsity regularization in addition to directional-derivative magnitude sparsity terms. Furthermore, the presented constrained, sparsity regularization framework includes pre-conditioning and algorithmic modifications that improve computational efficiency.

The 2D simulation studies conducted in this work focus on mapping out the parameter space with the goal of achieving accurate and stable image reconstruction from LAR data. The main conclusion from these studies is that the use of voxels substantially larger than the detector pixels is necessary and that pixel/voxel sparsity regularization, with the $\ell_{1}$-norm, combined with DTV terms is more effective than the use of DTV minimization alone. The 3D simulations demonstrate the impact of discretization error and are useful for determining the parameters of the L1-DTV min algorithm. With the L1-DTV min algorithm established, a processing pipeline for DE-DBT is developed using weighted subtraction of L1-DTV min reconstructed volumes from the low and high kV DBT acquisitions. The full image reconstruction algorithm is tested on a DE-DBT scan of the CIRS BR3D phantom with ICA objects. The results using L1-DTV show a dramatic reduction of depth blurring and that there is promise in using this algorithm to achieve quantitative ICA imaging with DE-DBT.

The L1-DTV min algorithm may prove useful for application to DBT for screening, but the parameters would have to be re-optimized based on relevant imaging tasks such as the detection of subtle microcalcifications or architectural distortions. As the focus in this work is on quantitative imaging, the optimal voxel size is large compared with the detector pixels, which may compromise detection tasks important for mammographic screening.

The presented application to CE-DBT is meant only as a demonstration of the potential application of L1-DBT using an inanimate physical phantom. As such, robustness against patient motion is not tested. The presented scan protocol, using two separate sweeps of the X-ray source, is also not appropriate for patient scanning because patient motion will likely cause inconsistency between the dual-energy DBT data sets. Future work will make use of a dual-layer detector that can acquire projections with two different spectral sensitivities simultaneously, thereby improving robustness against patient motion.^21^ Such an acquisition will also facilitate dual-energy sinogram decomposition.

Because the proposed algorithm relies on different forms of object sparsity, the performance of L1-DTV min is necessarily object-dependent. As a result, thorough testing on subjects of varying structural complexity is needed. However, the fact that the BR3D phantom does have a highly non-uniform background provides confidence that L1-DTV min can generalize well to other scanned subjects.

In addition to testing on different subjects, future work will focus on incorporating more accurate physics modeling in the image reconstruction and processing pipeline. The use of the image subtraction technique for dual-energy imaging does not address beam-hardening, and this is the next issue to be addressed in improving quantitative ICA imaging with DE-DBT. To accomplish this, the basis sinogram estimation method by Alvarez and Macovski^22^ will be adopted followed by image reconstruction with L1-DTV min. Accurate basis sinogram estimation requires knowledge of the DBT system spectral response for which we have been developing multiple calibration techniques.^23^^–^^25^ Scatter estimation and correction is another key component to developing quantitative ICA imaging, and again, we have developed tools to implement scatter estimation in DBT.^26^^,^^27^ With this additional processing in place, the post-processing background estimation should no longer be necessary; furthermore, reconstruction of the basis sinograms into basis images will allow the formation of images where the gray values have physical meaning such as density of the ICA or virtual monochromatic attenuation values.

## Data Availability

The code and data for the 2D simulations will be provided upon request. Please send an e-mail request to the contact author.
